# Ranking Power Spectra: A Proof of Concept

**DOI:** 10.3390/e21111057

**Published:** 2019-10-29

**Authors:** Xilin Yu, Zhenning Mei, Chen Chen, Wei Chen

**Affiliations:** 1 Center for Intelligent Medical Electronics (CIME), Fudan University, Shanghai 200433, China; 18210720171@fudan.edu.cn (X.Y.); 16210720037@fudan.edu.cn (Z.M.); 2 Human Phenome Institute, Fudan University, Shanghai 200433, China

**Keywords:** biomedical signal processing, order structure, spectral entropy

## Abstract

To characterize the irregularity of the spectrum of a signal, spectral entropy and its variants are widely adopted measures. However, spectral entropy is invariant under the permutation of the power spectrum estimations on a predefined grid. This erases the inherent order structure in the spectrum. To disentangle the order structure and extract meaningful information from raw digital signal, a novel analysis method is necessary. In this paper, we tried to unfold this order structure by defining descriptors mapping real- and vector-valued power spectrum estimation of a signal into a scalar value. The proposed descriptors showed its potential in diverse problems. Significant differences were observed from brain signals and surface electromyography of different pathological/physiological states. Drastic change accompanied by the alteration of the underlying process of signals enables it as a candidate feature for seizure detection and endpoint detection in speech signal. Since the order structure in the spectrum of physiological signal carries previously ignored information, which cannot be properly extracted by existing techniques, this paper takes one step forward along this direction by proposing computationally efficient descriptors with guaranteed information gain. To the best of our knowledge, this is the first work revealing the effectiveness of the order structure in the spectrum in physiological signal processing.

## 1. Introduction

Many real-world signals including physiological signals are irregular in some aspects. They are neither purely periodic nor can they be expressed by an analytic formula. The inherent irregularities imply the uncertainty during the evolution of the underlying process from which the signals are observed. The uncertainty enables information transfer but also limits the predictability of those signals. The unpredictability of signal in time domain makes researchers have to toil in frequency domain. Fourier transform (FT) bridges signals in original space (time domain) with their representations in dual space (frequency domain) by decomposing a signal satisfying some weak constraints into infinitely many periodic components, which has numerous applications in signal processing. 

For most of the real-world applications, finite samples drawn (usually evenly spaced) from a continuous random process cannot give us full information about the process’ evolution but only a discrete depiction. FT was adapted into discrete Fourier transform (DFT) for such scenarios [1]. Moreover, line spectrum wherein the total energy of the signal distributes on only few frequency components is rarely encountered among physiological signals due to the inherent irregularities therein.

To characterize the irregularity of digital signals in frequency domain, spectral entropy is introduced analogous to the Shannon entropy in information theory [2]. The estimations on the frequency grid are firstly divided by the total power, and then, a list of proxies in the form of probabilities whose sum is 1 is obtained. Then, the Shannon entropy formula, which is the negative sum of probability-weighted log probabilities, map those proxies into a quantity representing the irregularity of energy distribution on frequency domain. Under this perspective, a flat spectrum has maximal spectral entropy, and the spectrum of a single frequency signal has minimal spectral entropy, which is zero. Spectral entropy has been applied in diverse areas, including endpoint detection in speech segmentation [3] and spectrum sensing in cognitive radio [4]. Moreover, it has also served as the base of a famous inductive bias, maximum entropy [5], which is widely adopted for spectrum estimation of some kinds of physiological signals like electroencephalogram (EEG). 

Although spectral entropy is well-defined and can be computed efficiently by Fast Fourier Transform (FFT), it is difficult to relate spectral entropy with other interpretable properties of interest of original signal, especially when taking no account of the overwhelming endorsement from its counterpart (information entropy), which is the foundational concept in information theory which quantifies the uncertainty. Furthermore, it is apparent that the spectral entropy ignored the order information since the power estimations are arranged on the frequency grid with intrinsic partial order structure. Any permutations of these values on the grid yields a same spectral entropy, but obviously, the representations of those signals in time domain can look very different.

The motivation to incorporate the order information carried by the power spectrum is guided by the following belief. The normal operations of any system (biological/electromechanical, etc.) are impossible without the proper processing of information through some physical/chemical process. It could be the signaling between different modules within the system or the communications between the system as a whole and the external environment. Information transfers happening in those scenarios are accomplished with the help of carrier signals of particular forms with nontrivial structures in their spectra. Moreover, only limited frequency precision of the control and recognition of those signals is practical for real systems. Therefore, it is unreasonable for well-designed artificial systems or natural systems that have gone through long-term evolution to arrange the internal signals responsible for different functions close with each other in frequency domain within a certain time window. Otherwise, the efficient transfer of information could be degraded, and frequency divided multiplex [4] in modern communication systems can be considered as a living example of this belief.

Therefore, if we use power estimations on the frequency grid as proxies of the intensities of activities corresponding to those frequencies, it seems reasonable to infer that the energy distributed on neighboring rather than remote frequency grids is more likely caused by the very same function. The alpha band activities (8–13 Hz) which can be interrupted by visual perception tasks in human’s EEG is one of the examples. To sum up, we want to develop a metric to characterize the aforementioned structural irregularities of the power spectra, that is, how the frequency components of different intensities in a spectrum close to each other instead of what is captured in spectral entropy, which is how the intensities of frequency components are distributed no matter their locations in frequency domain. It was supposed to assign a larger value to a signal wherein the frequency components having similar intensities are distributed far apart from, rather than close to, each other. In addition, the similarities of intensities can be reflected (partially and heuristically) by the relative order of power estimates on discrete frequency grid. That is why the order information in the spectrum can shed new light on the structure aspects of signal and how the order information is incorporated into our analysis.

In this paper, we explore the effectiveness of the order information carried by the power spectra of signals. Given the motivation illustrated above, in Section 2 we provide details about our method. In Section 3 we present several use cases to justify the effectiveness of our preliminary approach and, more importantly, the promising potential to find some new research niche in the field of physiological signal processing. Finally, discussion about the limitations of our work and future directions are followed in Section 4.

## 2. Materials and Methods 

Given an equally spaced, real-valued digital signal s, we assume the length of s is an even number 2N, for simplicity. Then DFT is applied to s and a complex-valued vector $\hat{s}$ of dimension 2N is obtained as follows:

(1) $$\hat{s_{k}}=\sum_{i=0}^{2N-1}s_{i}\cdot e^{-j\cdot \frac{i\cdot k\cdot 2\pi }{2N}},\;k=0,1,\ldots ,2N-1$$

Due to the conjugate symmetry of $\hat{s}$, we take the square of the modulus of the first half of $\hat{s_{k}}$ and get $P_{s}\in R^{N}$. Thanks to the Parseval identity, the 1-norm of $P_{s}$ equals to the energy of s up to factor 1/2. Although $P_{s}$ has a dimension of energy instead of power, the constant factor having a dimension of time does not change the relative ordinal relations between its components. So we just use $P_{s}$ as the estimations of power on normalized frequency range [0,π], whereby the $k_{th}$ component of $P_{s}$ is the estimation of signal’s power on grid point (k−1)·π/N.

Now, let us assume again that every two components of $P_{s}$ are different from each other, so we can rank these N components without any ambiguity in ascending/descending order.

These grid points have an intrinsic partial order structure from low frequency range to high frequency range, so we get eigen-triple for these grid points:

(2) $$\left(\begin{array}{ccc} 1 & 2 & 3\\ \begin{array}{c} P_{s}\left(1\right)\\ \sigma \left(1\right) \end{array} & \begin{array}{c} P_{s}\left(2\right)\\ \sigma \left(2\right) \end{array} & \begin{array}{c} P_{s}\left(3\right)\\ \sigma \left(3\right) \end{array} \end{array}\begin{array}{cc} \ldots & N\\ \begin{array}{c} \ldots \\ \ldots \end{array} & \begin{array}{c} P_{s}\left(N\right)\\ \sigma \left(N\right) \end{array} \end{array}\right)$$

The first row indicates the grid points by their location on frequency range. The second row contains the corresponding power estimations. The third row contains the relative order of corresponding power estimation among all estimations, denoted by σ(·). Since no duplicated values in $P_{s}$ are assumed, ${\left\{\sigma \left(i\right)\right\}}_{i=1}^{N}$ will traverse number set {1,2,3,…,N}.

The first two rows of (2) are just a kind of representation of traditional power spectrum. Novelty lies in taking the order information, carried in the third row, into consideration. 

It should be noted that the first and the third row together have defined a permutation over the natural number set {1,2,3,…,N}, with its complete detail determined by s implicitly. Remember that spectral entropy is defined in a permutation-invariant way. Such an invariance must be broken down so as to disentangle the order information. Therefore, this permutation *per se* returns the long-overdue ladder to understand structural irregularities of signals under a new perspective.

**Figure 1:**
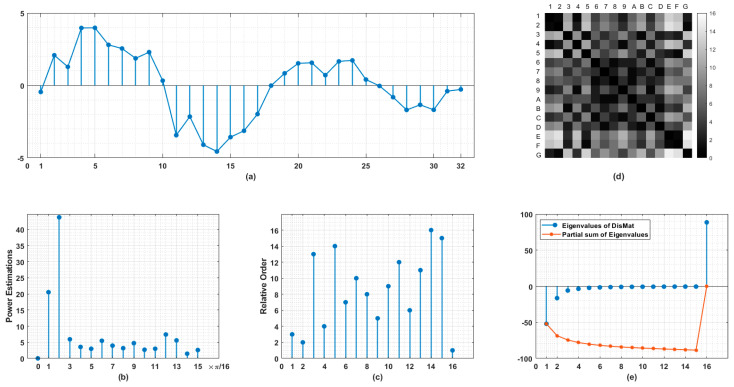
Schematic diagram of proposed method. (**a**) Original signal represented as equally spaced time series. (**b**) Power estimations on normalized frequency range [0,π], the number of points in (**b**) is just half of those in (**a**). (**c**) Relative orders of frequency components. The values on horizontal axis indicate the ranking of frequency components in descending order. The values on vertical axis indicate the location of corresponding frequency component in normalized range [0,π]. (**d**) Distance matrix (DisMat) of frequency components arranged in (**c**) whereby A-G represent 10–16th points. (**e**) Eigenvalues of DisMat and their partial sums. Note that DisMat’s trace identically equals to 0.

The sketch of our method is illustrated in Figure 1. Using the measurements in time domain (Figure 1a), the power estimations on normalized frequency grid with resolution determined by half the length of original signal are obtained (Figure 1b). By ranking these estimations in descending order, we arrange $\sigma_{\left(i\right)}^{-1}$ against i. As shown in Figure 1c, the first stem indicates the location on the frequency grid of the largest power component and so on. From (b) to (c), we are actually performing a nonlinear stretching while the order information of the spectrum is preserved and calibrated. Then a distance matrix M (in Figure 1d) is induced for every point pair. Here in (c) we define $M_{ij}=M_{ji}=\left|\sigma_{\left(i\right)}^{-1}-\sigma_{\left(j\right)}^{-1}\right|$.

So M is real-symmetric with trace identically equals to 0. The structural aspects of M are reflected in its eigenvalues (Figure 1e). Due to the sophisticated relationships between its entries, it is unwise to reshape such a high dimensional object with far lower degrees of freedom into a long vector for pattern recognition. In addition to the eigenvalues, a descriptor, named as *Circular Difference Descriptor* ($C_{i}D$), accounting for the total variation of the locations on frequency grids of frequency components having adjacent intensities is defined as follows, to a large extent, in a heuristic manner:

(3) $$C_{i}D_{N}=\frac{1}{N}\cdot \left(\left|\sigma_{\left(N\right)}^{-1}-\sigma_{\left(1\right)}^{-1}\right|+\sum_{i=1}^{N-1}\left|\sigma_{\left(i\right)}^{-1}-\sigma_{\left(i+1\right)}^{-1}\right|\right)$$

The first term makes Circular Difference veritable and endows $C_{i}D$ translational invariance instead of permutational invariance. 

Another heuristic descriptor is defined slightly different from $C_{i}D_{N}$, named as *Correspondence Difference Descriptor* ($C_{o}D$). It equals to the 1-norm of the difference of $\sigma_{\left(i\right)}^{-1}$ and i, aiming to characterize the difference between ${\left\{\sigma^{-1}\left(i\right)\right\}}_{i=1}^{N}$ and the perfectly ordered case where σ(i)=i,

(4) $$C_{o}D_{N}=\frac{1}{N}\cdot \sum_{i=1}^{N}\left|\sigma_{\left(i\right)}^{-1}-i\right|$$

**Figure 2:**
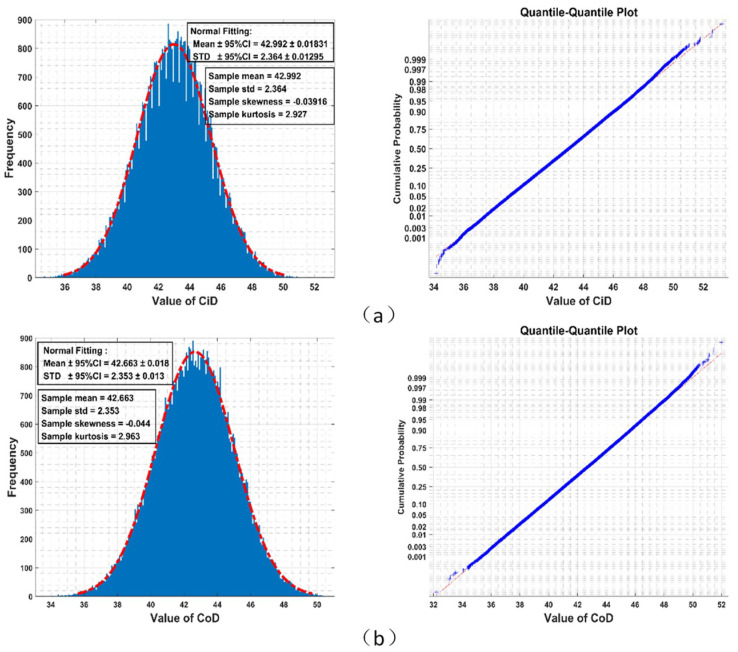
Given N=64, 640,000 times random generations of permutation over N letters yields an empirical distribution of Circular Difference Descriptor ($C_{i}D$) fitting normal distribution well (red dot dash line) in the left panel (STD: standard deviation, CI: confidence interval). The right panel is the quantile-quantile plot of this empirical distribution (blue cross) with theoretical value of normal distribution (red dot dash line). (**a**) Circular Difference Descriptor ($C_{i}D$); (**b**) Correspondence Difference Descriptor ($C_{o}D$).

Results from the Monte-Carlo simulation (shown in Figure 2) imply that the empirical distributions of $C_{i}D_{N}$ and $C_{o}D_{N}$ among all permutations could well be Gaussian. Although theoretical distributions of $C_{i}D_{N}$ and $C_{o}D_{N}$ must have bounded supports for finite N, they fit a bell-shaped curve very well, which in theory has unbounded support. 

Since permutational invariance of spectral entropy is broken herein, $C_{i}D$ and $C_{o}D$ actually encode the signal in different ways but both with guaranteed information gain with respect to spectral entropy. To be specific, given ${\left\{\sigma \left(i\right)\right\}}_{i=1}^{N}$ without ${\left\{P_{s}\left(i\right)\right\}}_{i=1}^{N}$, the corresponding $C_{i}D$ and $C_{o}D$ are fixed, but the distribution of ${\left\{P_{s}\left(i\right)\right\}}_{i=1}^{N}$ can form widely differed spectra. We take flat spectrum in (5) and line spectrum in (6) as examples:

(5) $$P_{s}\left(i\right)=k+\sigma_{\left(i\right)}^{-1}\cdot \varepsilon ,\;\varepsilon ↓0\;,\;k↑\frac{1}{N}$$

(6) $$P_{s}\left(\sigma_{\left(1\right)}^{-1}\right)=1-\frac{N\left(N-1\right)}{2}\cdot \varepsilon ,\;P_{s}\left(\sigma_{\left(i\right)}^{-1}\right)=\varepsilon \cdot i,\;i\neq 1,\;\varepsilon ↓0$$

The corresponding spectral entropy values vary from infinitesimal (in (5)) to maximum possible (in (6)). On the contrary, given ${\left\{P_{s}\left(i\right)\right\}}_{i=1}^{N}$, any permutation on it yields an exactly same spectral entropy, as mentioned before, but the corresponding $C_{i}D$ and $C_{o}D$ will absolutely transverse all possible values.

**Figure 3:**
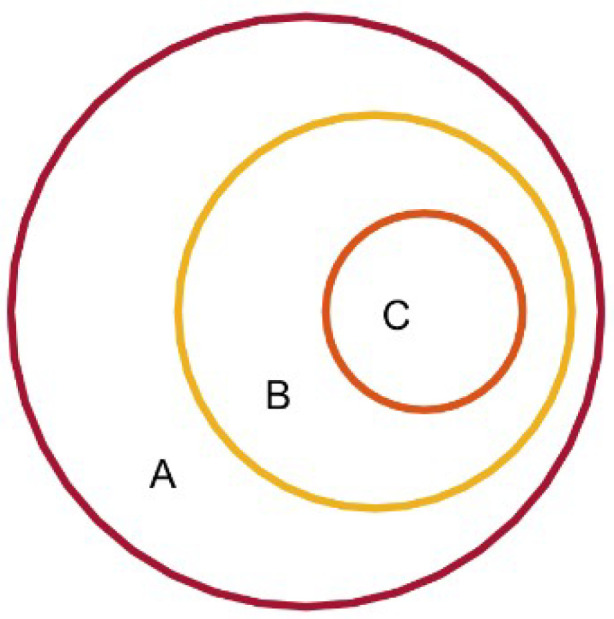
The relationship between spectral entropy and the order information.

The relationship between spectral entropy and the proposed descriptors is illustrated in Figure 3. The set A denotes full space of signals’ spectra whereby for each ${\left\{P_{s}\left(i\right)\right\}}_{i=1}^{N}$ no duplicate value exists for its sub-components $P_{s}\left(i\right)\neq P_{s}\left(j\right),\forall i\neq j\in \left\{1,2,\ldots ,N\right\}$, which is an assumption made by us for simplicity and with only minimal loss of generality. Signals in the set B have the same spectral entropy, denoted by $SE_{0}$. The following conditions need to be satisfied for signals in C:

(7) $$SE\{{\left\{P_{i}\left(k\right)\right\}}_{k=1}^{N}\}=SE\{{\left\{P_{j}\left(k\right)\right\}}_{k=1}^{N}\}=SE_{0},i\neq j$$

(8) $$\sigma \{{\left\{P_{i}\left(k\right)\right\}}_{k=1}^{N}\}=\sigma \{{\left\{P_{j}\left(k\right)\right\}}_{k=1}^{N}\}$$

(9) $${\left\{P_{i}\left(k\right)\right\}}_{k=1}^{N}\neq {\left\{P_{j}\left(k\right)\right\}}_{k=1}^{N}\;\mathrm{under}\;\mathrm{any}\;\mathrm{permutation}$$

Spectral entropy operator is denoted by SE{·}, and $\sigma \{{\left\{P_{i}\left(k\right)\right\}}_{k=1}^{N}\}$ denotes the *rank vector* of ${\left\{P_{i}\left(k\right)\right\}}_{k=1}^{N}$. For example, if we have $P_{i}\left(k\right)>P_{i}\left(k+1\right),\forall k\in \left\{1,2,\ldots ,N-1\right\}$, then we will have $\sigma \{{\left\{P_{i}\left(k\right)\right\}}_{k=1}^{N}\}=\left[1,2,3,\ldots ,N\right]$.

Since all members in C are with a same *rank vector*, we can obtain many different counterparts of C which share this spectral entropy value with it by a certain permutation on the arrangement of ${\left\{P_{i}\left(k\right)\right\}}_{k=1}^{N}$. Since there are (N!−1) different permutations other than the identical permutation, the following relationship is obtained:

(10) $$B=C_{1}\cup C_{2}\cup \cdots \cup C_{N!}$$

(11) $$C_{i}\cap C_{j}=\emptyset ,\;i=1,2,\cdots ,\;N,\;j=1,2,\cdots ,\;N,i\neq j$$

Until now, we get a coverage of B by N! disjoint subsets. Members in the same subset share a specific spectral entropy value, a same *rank vector,* and cannot be transformed to be identical to each other by simply rearranging their sub-components.

Given only the value of spectral entropy ($SE=SE_{0}$) without *rank vector*, we can localize the signal in A to B. Given $\sigma \{{\left\{P\left(k\right)\right\}}_{k=1}^{N}\}$, the location will be more accurate (to one of many Cs in B). From this perspective, we can distinguish signals which have completely different order structures with the same spectral entropy.

If no a priori about the signals’ spectra is available, then the equiprobable distribution of ${\left\{\sigma \left(i\right)\right\}}_{i=1}^{N}$ is substantially and implicitly pre-assumed. Then under such circumstance, the so-called Kullback–Liebler Divergence (KLD) which is a widely used method to measure the difference between two probability distributions is adopted to illustrate the advantage when using the proposed descriptors. KLD between the proposed descriptors and spectral entropy as different coding schemes having probability distribution $p_{1}\left(\cdot \right)$ and $p_{2}\left(\cdot \right)$ are always nonnegative [6], no matter the direction (KLD is lack of symmetry). KLD between two distributions $p_{1}\left(x\right)$ and $p_{2}\left(x\right)$ is defined as follows:

(12) $$\begin{array}{c} KL(p_{1}\|p_{2})=\int p_{1}\left(x\right)log\frac{p_{1}\left(x\right)}{p_{2}\left(x\right)}dx\geq 0\\ KL(p_{2}\|p_{1})=\int p_{2}\left(x\right)log\frac{p_{2}\left(x\right)}{p_{1}\left(x\right)}dx\geq 0 \end{array}$$

Such a property is welcomed since it guarantees the nonnegative information gain when using both spectral entropy and the proposed descriptors instead of only one of them. In other words, the representation will be more informative with a combination of our proposed descriptors and spectral entropy.

As for the distance matrix with its entries $M_{ij}$ representing the *distance* or *similarity* between point i and j, distance measures other than the absolute difference can be applied on $\sigma_{\left(i\right)}^{-1}$ and $\sigma_{\left(j\right)}^{-1}$ to form different distance matrices. Given any distance measure, a topology is induced on this finite set ${\left\{P_{s}\left(i\right)\right\}}_{i=1}^{N}$, based on the coarse-grained, discrete-valued rankings among them, and certainly, more order information is unrevealed yet. For example, $C_{i}D$ is just the circular difference of the first sub-diagonal line of M, captures only a portion of full information.

To sum up, by ranking power estimations of signal on a discrete frequency grid, an interesting picture of order structure carried by signal’s spectrum is obtained.

## 3. Results

In this section we provide several use cases to show the effectiveness of order information carried by signal’s power spectrum in physiological signal processing.

### 3.1. Surface Electromyography (sEMG)

**Figure 4:**
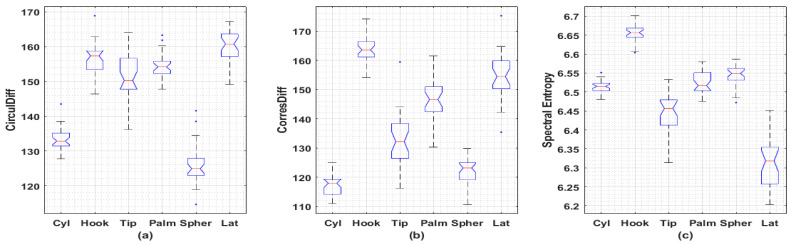
Boxplots of irregularity metrics obtained from sEMG signals collected from 6 different actions. (**a**) Circular Difference Descriptor; (**b**) Correspondence Difference Descriptor; (**c**) Spectral Entropy. Totally, there are 5 subjects (3 females and 2 males) involved in this dataset. Each subject repeated 30 times for each action with 2 channels’ EMG signals recorded. Each recording is of length 6 s under sampling frequency 500 Hz (3000 data points). Only the latest 2048 data points for each recording are used to generate these metrics. Female 3, channel 2 is used here with remaining boxplots left in the Supplementary Information. Significant differences (*p* < 0.001) between sample medians are observed among inter-subset Wilcoxon rank sum tests, except for Hook-vs-Tip (*p* = 0.0017), Hook-vs-Palm (*p* = 0.0378), Hook-vs-Tip (*p* = 0.0042), Tip-vs-Palm (*p* = 0.0138) in CirculDiff’s comparisons and Cyl-vs-Palm (*p* = 0.2340), Palm-vs-Spher (*p* = 0.0117) in Spectral Entropy’s comparisons. Significant differences (*p* < 0.01) between sample medians are observed among Kruskal–Wallis test with Bonferroni’s correction, except for Spher-vs-Cyl (*p* = 1.000), Tip-vs-Palm (*p* = 1.000), Hook-vs-Tip (*p* = 0.467), Hook-vs-Palm (*p* = 1.000), Palm-vs-Lat (*p* = 0.134), Hook-vs-Lat (*p* = 1.000) in CirculDiff’s comparisons; Spher-vs-Cyl (*p* = 1.000), Spher-vs-Tip (*p* = 0.288), Tip-vs-Palm (*p* = 0.171), Palm-vs-Lat (*p* = 1.000), Hook-vs-Lat (*p* = 0.704) in CorresDiff’s comparisons; Lat-vs-Tip (*p* = 0.294), Tip-vs-Cyl (*p* = 0.044), Cyl-vs-Palm (*p* = 1.000), Cyl-vs-Spher (*p* = 0.240), Palm-vs-Spher (*p* = 1.000), Spher-vs-Hook (*p* = 0.016) in Spectral Entropy’s comparisons. (Cyl: Cylindrical, for holding cylindrical tools; Hook: for supporting a heavy load; Tip: for holding small tools; Palm: Palmar, for grasping with palm facing the object; Spher: Spherical, for holding spherical tools; Lat: Lateral: for holding thin, flat objects; CirculDiff: Circular Difference Descriptor; CorresDiff: Correspondence Difference Descriptor).

It was found the proposed descriptors may be able to distinguish sEMG signals collected under different actions. A publicly available dataset containing sEMG recordings from three females and two males acting six different actions is involved in the analysis [7]. Wilcoxon rank sum test and Kruskal–Wallis test with Bonferroni’s correction are used to compare the medians of each class. A representative example is given in Figure 4 with statistical significance achieved between the medians among most of comparisons. As for full comparison, Supplementary Information contains all comparisons for remaining subjects.

### 3.2. Electroencephalogram (EEG)

**Figure 5:**
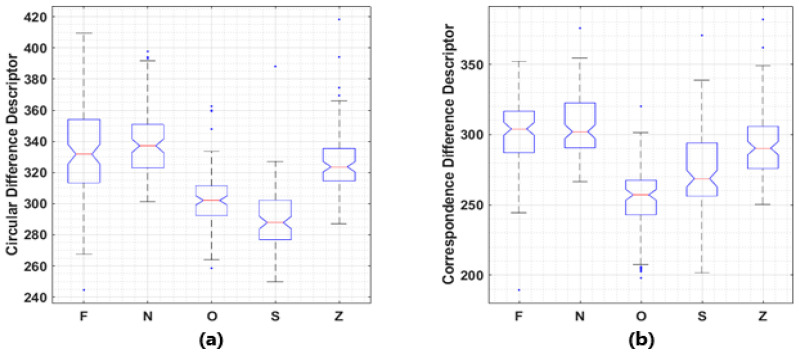
Boxplots of the value distributions of two descriptors. (**a**) Circular Difference Descriptor; (**b**) Correspondence Difference Descriptor. Bonn seizure dataset consists EEG recordings collected from 5 healthy volunteers with eyes open (Z) or closed (O) and 5 patients during ictal period (S), inter-ictal period in epileptogenic zone (F), or hippocampal formation of the opposite hemisphere (N). Another classification criterion is healthy EEG (OZ), inter-ictal period activities (FN), and ictal period activities (S). Significant differences (*p* < 0.001) between sample medians are observed among inter-subset Wilcoxon rank sum tests, except for F-vs-N (*p* = 0.238) and F-vs-Z (*p* = 0.002) in Correspondence Difference Descriptor’s comparisons, F-vs-N (for *p* = 0.158) in Circular Difference Descriptor’s comparisons.

It was also found that the proposed descriptors may be able to distinguish brain signals under different pathological states. A publicly available dataset, Bonn Seizure Dataset, which is widely used as materials for brain signal related pattern recognition and machine learning tasks is employed [8]. In this dataset, 5 subsets contain 100 recordings each with identical length, sampling frequency and other conditions, collected under different pathological states. Rank sum test used in 3.1 is performed. Significant (*p* < 0.001) differences between the medians of the values of proposed descriptors corresponding to these 5 subsets were observed in most of the cases, with boxplots given in Figure 5. 

### 3.3. Speech Signal

When N is fixed and performing the operator defined in (3) and (4) on moving window mounted on a long signal, we are able to unfold the structural irregularities of signal in a finer time resolution, and thus, change point detection is possible. 

**Figure 6:**
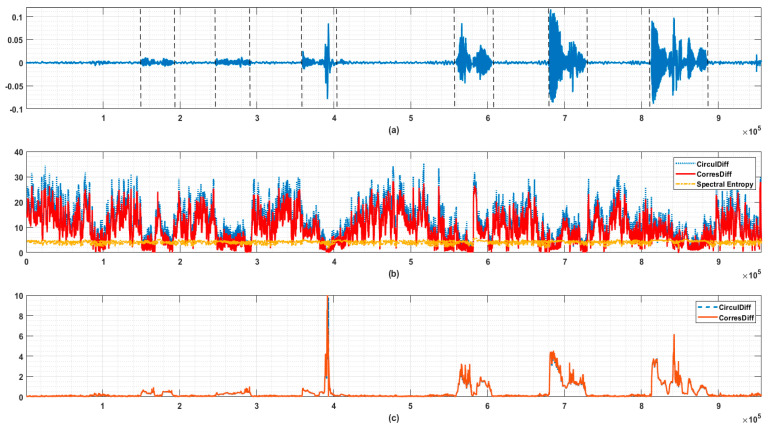
Use case of endpoint detection in speech signal. (**a**) Original speech signal. The starts and end points of syllables are indicated by dashed lines. (**b**) Circular Difference Descriptor, Correspondence Difference Descriptor, and Spectral Entropy of signal (**a**). The window length is 1024, and step length is 128 here. The energy threshold q is set to be 0.9 here. It is obvious that the proposed descriptors are more sensitive to the appearance of syllables (reduced irregularity implies possible formant caused) than spectral entropy. (**c**) Monitoring value taking local energy into consideration (let the standard deviation of last 1024 points be LE here). The values on y-axis are calculated by $\log_{10}\left(1+\mathrm{LE}\right)/\log_{10}\left(\mathrm{Descriptor}\right)$ (CirculDiff: Circular Difference Descriptor; CorresDiff: Correspondence Difference Descriptor).

In Figure 6 we provide an example of endpoint detection in human speech signals. It can be seen that the start points and stop points of syllables are accompanied by the steep increase/decrease of descriptors’ values. In this example, we also found that the descriptors defined in (3) and (4), which are purely based on order information, as opposed to spectral entropy, which has nothing to do with order information, could become noise vulnerable in some problems. This is due to the amplitudes of ${\left\{P_{s}\left(i\right)\right\}}_{i=1}^{N}$ are barely removed after transforming into ${\left\{\sigma \left(i\right)\right\}}_{i=1}^{N}$. Consider such a case where a large portion of ${\left\{P_{s}\left(i\right)\right\}}_{i=1}^{N}$ only accounts for a negligible portion of total energy, then, their relative order can vary drastically because of possible noise and so can the descriptors’ values. However, it seems unreasonable to deem the structure of signal must have changed accordingly.

Therefore, a simple thresholding segmentation trick of total variance, similar to what is usually adopted in principal component analysis is used in this case. The descriptors are calculated based on the first L components ${\left\{\sigma_{\left(i\right)}^{-1}\right\}}_{i=1}^{L}$ whereby L is defined as follows:

(13) $$L=\min\left\{l\;|\sum_{i=1}^{l}P_{s}\left(\sigma_{\left(i\right)}^{-1}\right)\geq q\cdot \left(\sum_{i=1}^{N}P_{s}\left(i\right)\right)\right\}$$

The q∈(0, 1] is a tunable parameter selecting *L* largest frequency components accounting for just above a preset portion of total energy. This trick improves the robustness against wide-band weak noise but removes some welcomed properties. Possible modifications of naive descriptors proposed here will be discussed later. 

### 3.4. Amplitude-Integrated EEG (aEEG)

Another example validates the effectiveness of the proposed method by revealing the temporal evolution of physiological process is founded in the analysis of aEEG [9]. aEEG is a kind of condensed sketch of long-term EEG recording. It was believed to be able to reflect long term trends of brain activities in a horizon suitable for visual inspection and evaluation. It has been widely used for seizure detection in neonates, brain disorder evaluation, etc. 

**Figure 7:**
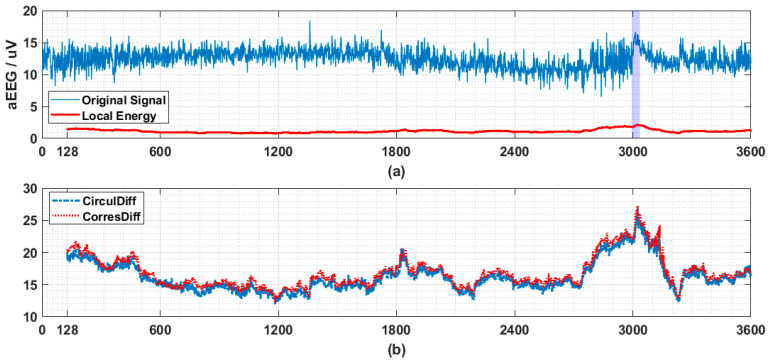
The application of proposed descriptors on seizure detection. (**a**) Amplitude-integrated EEG (aEEG) tracing contains a seizure event (indicated by purple bar from 2996th to 3036th s) with its local energy (here standard deviation of last 128 points, referred as LE is used). The original multichannel EEG signals (23 channels with sampling frequency being 256 Hz) are drawn from patient 1, record 3 from CHB-MIT dataset and consist of a one hour recording. The channel 7 (C3-P3) is transformed by recommended pipeline into aEEG. Every second’s data points (256 data points) are compressed into 2 data points (upper bound and lower bound each). So totally 3600 × 2 data points are obtained. Here we use only upper bound, and similar results have also been observed for lower bound data. (**b**) Circular Difference Descriptor and Correspondence Difference Descriptor monitoring (calculated based on last 128 data points in (**a**)). The step length is fixed to be 1 here, which means that after obtaining every new point, the values of two descriptors will be updated to provide the finest and most sensitive anomaly monitoring. The values on y-axis are calculated by $\log_{10}\left(1+\mathrm{LE}\right)/\log_{10}\left(\mathrm{Descriptor}\right)$. Drastic change accompanied by the onset of seizure (a high value first and then a steep decresing) is observed in both of the descriptors (CirculDiff: Circular Difference Descriptor; CorresDiff: Correspondence Difference Descriptor).

In Figure 7 a segment of EEG drawn from CHB-MIT dataset [10] is transformed into aEEG first and then similar analysis used in Section 3.3 is adopted. Ictal period is indicated by colored bar.

## 4. Discussion

Order structure of signal’s spectrum is revealed by simply ranking the power estimations. Several use cases justify that taking that order structure into consideration could contribute valuable information to the processing of physiological signals. The possible applications include serving as candidate features for pattern recognition among signals, change point detection in process tracking for anomaly detection and many more.

The permutation of length *N* defined by rankings of power estimations on frequency grid has huge capacity (N!). Although in practice it is not necessarily that these N! different ordinal patterns are equiprobable, the proved information gain under such an assumption is still hoped to be found in practice. An established metric, *permutation entropy* is based on ranking consecutive measurements in time domain and doing statistics among a sufficient number of segments [11]. The length of such segments must be small otherwise the density estimation will be impractical for time series of reasonable length. Our method delves into the order structure of signal’s representation in dual space (frequency domain) instead of original space (time domain). Every point in the dual space is bridged to all points in the time domain through FT, so no one-to-one correspondence exists between original measurements and mapped points in the proposed method. This is also an important distinction.

The proposed descriptors in their original forms could be noise vulnerable, but they can be modified using techniques include but not limit what is used here. In practice, we observed high correlation between $C_{i}D$ and $C_{o}D$, and one could outperform another at times. In addition, the pairwise distances in the distance matrix in Figure 1d can be induced in a way other than that used here. Anyway, more fruitful and distinguishable features can be extracted along this way from such a representation with large capacity. 

As for future research, we have several proposals.

The first is to establish relationships between the order information given by a recorded digital signal of length 2*N* and that of its sub-signals, obtained by (nonuniformly) down-sampling these 2*N* points. Uniformly down-sampling is equivalent to folding the power spectrum. Situations will be more sophisticated under nonuniform cases (include but not limit to evolving/truncating case where the length of signal is ever-increasing), but usually a more flat spectrum with lower frequency resolution is produced. The original signal with its sub-signals together could provide an informative and hierarchical object of study.

The second is to develop distance measures other than the absolute difference of ranks used here. By incorporating both the discrete-valued ranks and the continuous-valued power estimations, parameters more robust to broad band noise could be anticipated. Furthermore, could ‘ranking’ of power spectrum of a continuous function (signal) be possible in some sense?

The third is about the topology induced from the distance matrix. The distance matrix in Figure 1d or the distance defined by possible modified measures, as mentioned above, whereby block structures frequently occur, provides full neighborhood information of *N* points on frequency grid. Given such information, could we find some relations with the eigenvalues of DisMat (with possible modification mentioned in the second point) with some properties of interest of original signal? Despite that, we can also calculate so-called persistent homology—a dominant methodology usually referred as synonym of topological data analysis (TDA)—of these *N* points by computing a series of simplicial complexes with their topological invariants [12] and get topological description of signal’s power spectrum. That means the order information in spectrum enables a nontrivial embedding method of data points with temporal structure. Such an embedding method is different from the famous delay-embedding [13], which is an operation performed in signal’s original space rather than dual space. Delay-embedding could be vulnerable to short and noisy process. A messy point cloud could provide nothing except for ‘topological noises’. However, by ranking power spectrum, the data points are arranged in an organized way, and the application of TDA can be free from such pitfalls encountered in delay-embedding.

In conclusion, order structures of physiological signals’ power spectra are almost neglected in existing methods, but they are not meaningless. On the contrary, such structures could provide a unique perspective to understand the intrinsic properties of physiological processes.

## Supplementary Materials

Full intra-subject comparison of two descriptors across six different actions are available online at https://www.mdpi.com/1099-4300/21/11/1057/s1.
