# Supplementary material for: Ranking Power Spectra: A Proof of Concept
Source: Entropy (Basel). 2019 Oct 29;21(11):1057. doi: 10.3390/e21111057 (PMC7514361; doi:10.3390/e21111057)
Supplement: Supplementary file 1 [file entropy-21-01057-s001.pdf]

Supplementary Information

Full intra-subject comparison of two descriptors across six different actions

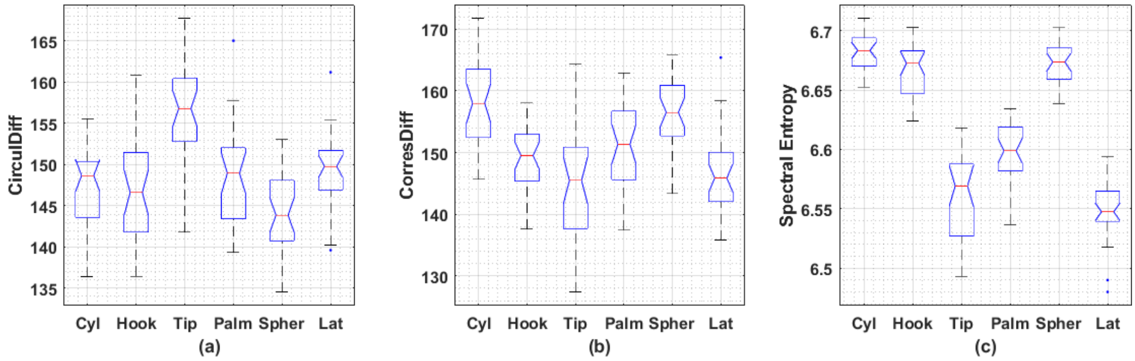

Figure S1. Comparisons of female one, channel one. Cases failed to pass Wilcoxon rank sum tests (13/45, confidence level is set to be 0.05): Cyl-vs-Hook (p = 0.4204), Cyl-vs-Palm (p = 0.5997), Cyl-vs-Lat (p = 0.1297), Hook-vs-Palm (p = 0.2519), Hook-vs-Spher (p = 0.1882), Palm-vs-Lat (p = 0.4733) in (**a**); Cyl-vs-Spher (p = 0.5395), Hook-vs-Tip (p = 0.0555), Hook-vs-Palm (p = 0.3255), Hook-vs-Lat (p = 0.0850), Tip-vs-Lat (p = 0.4918) in (**b**); Hook-vs-Spher (p = 0.3329), Tip-vs-Lat (p = 0.3042) in (**c**). Cases failed to pass Kruskal-Wallis test with Bonferroni's correction (25/45, confidence level is set to be 0.01): Spher-vs-Hook (p = 1.000), Spher-vs-Cyl (p = 0.455), Spher-vs-Palm (p = 0.104), Spher-vs-Lat (p = 0.010), Hook-vs-Cyl (p = 1.000), Hook-vs-Palm (p = 1.000), Hook-vs-Lat (p = 0.861), Cyl-vs-Palm (p = 1.000), Cyl-vs-Lat (p = 1.000), Palm-vs-Lat (p = 1.000) in (a); Tip-vs-Lat (p = 1.000), Tip-vs-Hook (p = 1.000), Tip-vs-Palm (p = 0.291), Lat-vs-Hook (p = 1.000), Lat-vs-Palm (p = 0.568), Hook-vs-Palm (p = 1.000), Palm-vs-Spher (p = 0.061), Palm-vs-Cyl (p = 0.017), Spher-vs-Cyl (p = 1.000) in (b); Lat-vs-Tip (p = 1.000), Lat-vs-Palm (p = 0.104), Tip-vs-Palm (p = 0.458), Hook-vs-Spher (p = 1.000), Hook-vs-Cyl (p = 1.000), Spher-vs-Cyl (p = 1.000) in (c).

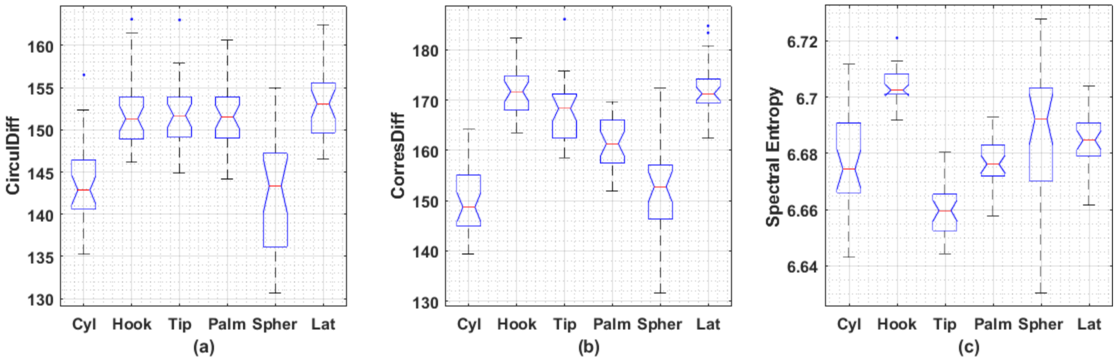

Figure S2. Comparisons of female one, channel two. Cases failed to pass Wilcoxon rank sum tests (6/45, confidence level is set to be 0.05): Cyl-vs-Tip (p = 0.4247), Hook-vs-Spher (p = 0.2116), Hook-vs-Lat (p = 0.9117), Spher-vs-Lat (p = 0.4204) in (a); Hook-vs-Spher (p = 0.1413) in (b); Tip-vs-Palm (p = 0.2707) in (c). Cases failed to pass Wilcoxon rank sum tests (6/45, confidence level is set to be 0.05): Cyl-vs-Tip (p = 0.4247), Hook-vs-Spher (p = 0.2116), Hook-vs-Lat (p = 0.9117), Spher-vs-Lat (p = 0.4204) in (**a**); Hook-vs-Spher (p = 0.1413) in (**b**); Tip-vs-Palm (p = 0.2707) in (**c**). Cases failed to pass Kruskal-Wallis test with Bonferroni's correction (22/45, confidence level is set to be 0.01): Tip-vs-Cyl (p = 1.000), Tip-vs-Hook (p = 0.016), Tip-vs-Lat (p = 0.014), Cyl-vs-Hook (p = 0.153), Cyl-vs-Lat (p = 0.141), Hook-vs-Lat (p = 1.000), Hook-vs-Spher (p = 1.000), Hook-vs-Palm (p = 0.136), Lat-vs-Spher (p = 1.000), Lat-vs-Palm (p = 0.147), Spher-vs-Palm (p = 1.000) in (a); Tip-vs-Cyl (p = 0.033), Cyl-vs-Palm (p = 1.000), Spher-vs-Hook (p = 1.000), Spher-vs-Lat (p = 0.675), Hook-vs-Lat (p = 1.000) in (b); Palm-vs-Tip (p =

1.000), Palm-vs-Lat (p = 0.018), Tip-vs-Lat (p = 0.186), Lat-vs-Cyl (p = 0.294), Cyl-vs-Spher (p = 0.266), Spher-vs-Hook (p = 0.901) in (**c**). .

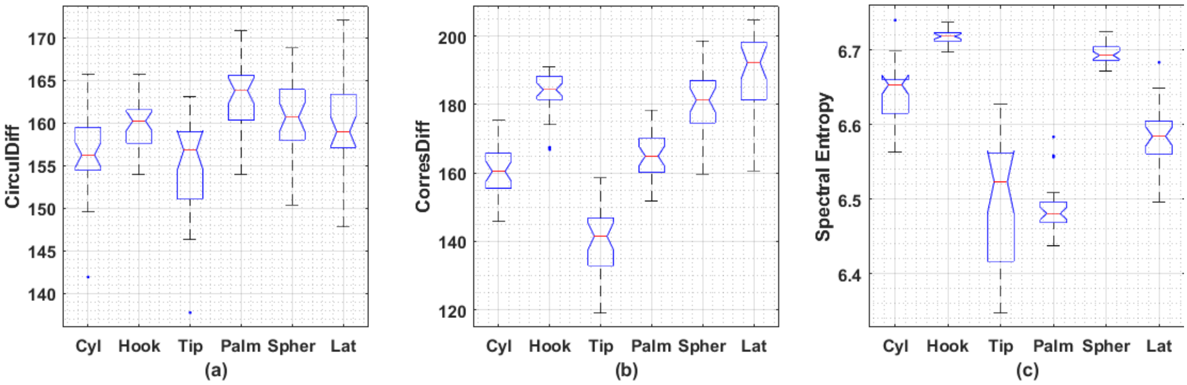

Figure S3. Comparisons of female two, channel one. Cases failed to pass Wilcoxon rank sum tests (12/45, confidence level is set to be 0.05): Cyl-vs-Spher (p = 0.8303), Hook-vs-Tip (p = 0.8418), Hook-vs-Palm (p = 0.9000), Hook-vs-Lat (p = 0.3711), Tip-vs-Palm (p = 0.9352), Tip-vs-Lat (p = 0.2340), Palm-vs-Lat (p = 0.3042) in (**a**); Cyl-vs-Spher (p = 0.2838), Hook-vs-Lat in (**b**); Cyl-vs-Palm (p = 0.9941), Cyl-vs-Spher (p = 0.1224), Cyl-vs-Lat (p = 0.0850), Spher-vs-Lat (p = 0.4204) in (**c**). Cases failed to pass Kruskal-Wallis test with Bonferroni's correction (21/45, confidence level is set to be 0.01): Cyl-vs-Spher (p = 1.000), Tip-vs-Palm (p = 1.000), Tip-vs-Hook (p = 1.000), Tip-vs-Lat (p = 1.000), Palm-vs-Hook (p = 1.000), Palm-vs-Lat (p = 1.000), Hook-vs-Lat (p = 1.000) in (a); Cyl-vs-Spher (p = 1.000), Cyl-vs-Palm (p = 0.017), Spher-vs-Palm (p = 0.189), Palm-vs-Tip (p = 0.066), Tip-vs-Hook (p = 1.000), Tip-vs-Lat (p = 0.861), Hook-vs-Lat (p = 1.000) in (b); Tip-vs-Palm (p = 0.027), Palm-vs-Cyl (p = 1.000), Palm-vs-Lat (p = 1.000), Palm-vs-Spher (p = 0.568), Cyl-vs-Lat (p = 1.000), Cyl-vs-Spher (p = 1.000), Lat-vs-Spher (p = 1.000) in (**c**).

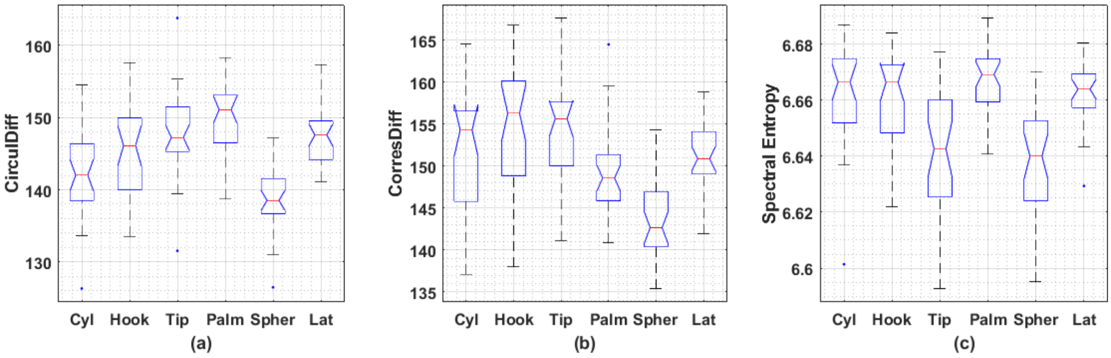

Figure S4. Comparisons of female two, channel two. Cases failed to pass Wilcoxon rank sum tests (17/45, confidence level is set to be 0.05): Hook-vs-Tip (p = 0.1958), Hook-vs-Lat (p = 0.2838), Tip-vs-Palm (p = 0.0555), Tip-vs-Lat (p = 0.5298) in (**a**); Cyl-vs-Hook (p = 0.2009), Cyl-vs-Tip (p = 0.3555), Cyl-vs-Palm (p = 0.0657), Cyl-vs-Lat (p = 0.1494), Hook-vs-Tip (p = 0.5011), Palm-vs-Lat (p = 0.0690) in (**b**); Cyl-vs-Hook (p = 0.6414), Cyl-vs-Palm (p = 0.4204), Cyl-vs-Lat (p = 0.6414), Hook-vs-Palm (p = 0.2340), Hook-vs-Lat (p = 0.9941), Tip-vs-Spher (p = 0.4035), Palm-vs-Lat (p = 0.1260) in (**c**). Cases failed to pass Kruskal-Wallis test with Bonferroni's correction (29/45, confidence level is set to be 0.01): Spher-vs-Cyl (p = 0.554), Cyl-vs-Hook (p = 0.671), Cyl-vs-Lat (p = 0.030), Hook-vs-Lat (p = 1.000), Hook-vs-Tip (p = 1.000), Hook-vs-Palm (p = 0.058), Lat-vs-Tip (p = 1.000), Lat-vs-Palm (p = 1.000), Tip-vs-Palm (p = 1.000) in (a); Spher-vs-Palm (p = 0.026), Palm-vs-Lat (p = 1.000), Palm-vs-Cyl (p = 0.955), Palm-vs-Tip (p = 0.167), Palm-vs-Hook (p = 0.070), Lat-vs-Cyl (p = 1.000), Lat-vs-Tip (p = 1.000), Lat-vs-Hook (p = 1.000), Cyl-vs-Tip (p = 1.000), Cyl-vs-Hook (p = 1.000), Tip-vs-Hook (p = 1.000) in (b); Spher-vs-Tip (p = 1.000), Tip-vs-Hook (p = 0.021), Tip-vs-Lat (p = 0.012), Hook-vs-Lat (p = 1.000), Hook-vs-Cyl (p = 1.000), Hook-vs-Palm (p = 1.000), Lat-vs-Cyl (p = 1.000), Lat-vs-Palm (p = 1.000), Cyl-vs-Palm (p = 1.000) in (**c**).

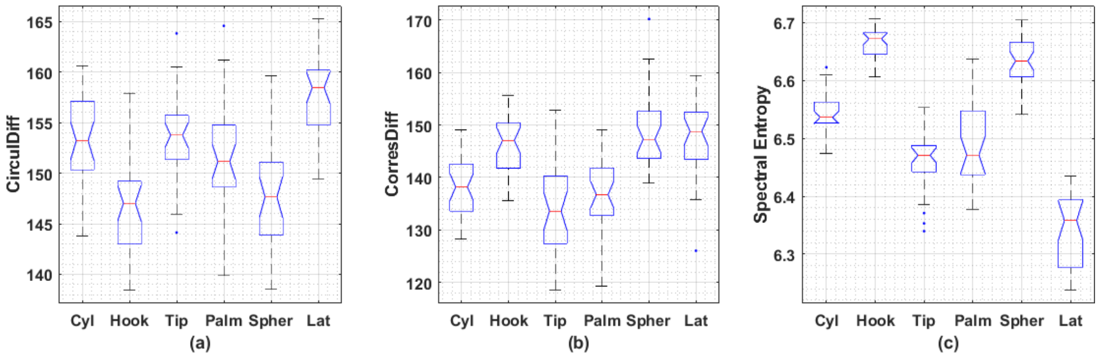

Figure S5. Comparisons of female three, channel one. Cases failed to pass Wilcoxon rank sum tests (10/45, confidence level is set to be 0.05): Cyl-vs-Tip (*p* = 0.6735), Cyl-vs-Palm (*p* = 0.2062), Hook-vs-Spher (*p* = 0.3112), Tip-vs-Palm (*p* = 0.0575) in (**a**); Cyl-vs-Palm (*p* = 0.4204), Hook-vs-Spher (*p* = 0.4643), Hook-vs-Lat (*p* = 0.5201), Tip-vs-Palm (*p* = 0.2707), Spher-vs-Lat (*p* = 0.7506) in (**b**); Tip-vs-Palm (*p* = 0.3790) in (**c**). Cases failed to pass Kruskal-Wallis test with Bonferroni's correction (20/45, confidence level is set to be 0.01): Hook-vs-Spher (*p* = 1.000), Hook-vs-Palm (*p* = 0.017), Spher-vs-Palm (*p* = 0.301), Palm-vs-Cyl (*p* = 1.000), Palm-vs-Tip (*p* = 1.000), Cyl-vs-Tip (*p* = 1.000), Cyl-vs-Lat (*p* = 0.030), Tip-vs-Lat (*p* = 0.119) in (a); Tip-vs-Palm (*p* = 1.000), Tip-vs-Cyl (*p* = 1.000), Palm-vs-Cyl (*p* = 1.000), Hook-vs-Lat (*p* = 1.000), Hook-vs-Spher (*p* = 1.000), Lat-vs-Spher (*p* = 1.000) in (b); Lat-vs-Tip (*p* = 0.052), Tip-vs-Palm (*p* = 1.000), Tip-vs-Cyl (*p* = 0.039), Palm-vs-Cyl (*p* = 0.461), Cyl-vs-Spher (*p* = 0.023), Spher-vs-Hook (*p* = 1.000) in (**c**).

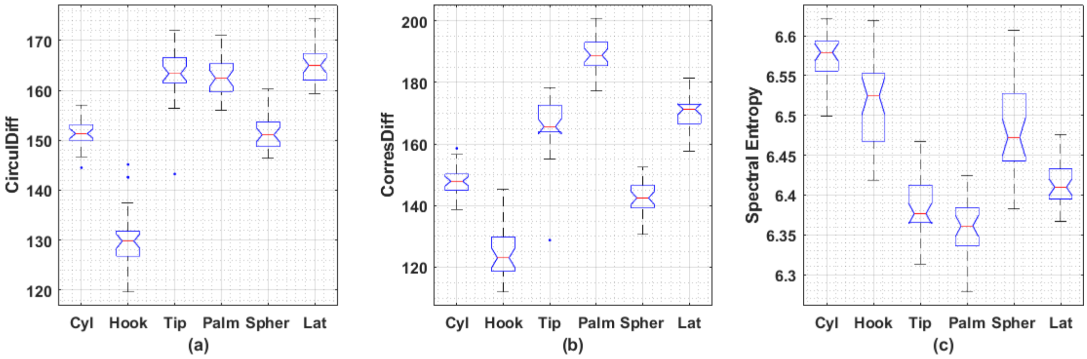

Figure S6. Comparisons of male one, channel one. Cases failed to pass Wilcoxon rank sum tests (4/45, confidence level is set to be 0.05): Cyl-vs-Spher (*p* = 0.7394), Tip-vs-Palm (*p* = 0.2282), Tip-vs-Lat (*p* = 0.5011) in (**a**); Tip-vs-Lat (*p* = 0.1297) in (**b**). Cases failed to pass Kruskal-Wallis test with Bonferroni's correction (16/45, confidence level is set to be 0.01): Hook-vs-Cyl (*p* = 0.012), Cyl-vs-Spher (*p* = 1.000), Palm-vs-Tip (*p* = 1.000), Palm-vs-Lat (*p* = 1.000), Tip-vs-Lat (*p* = 1.000) in (a); Hook-vs-Spher (*p* = 0.149), Spher-vs-Cyl (*p* = 1.000), Cyl-vs-Tip (*p* = 0.010), Tip-vs-Lat (*p* = 1.000), Lat-vs-Palm (*p* = 0.038) in (**b**); Palm-vs-Tip (*p* = 1.000), Palm-vs-Lat (*p* = 0.026), Tip-vs-Lat (*p* = 0.895), Lat-vs-Spher (*p* = 0.073), Spher-vs-Hook (*p* = 1.000), Hook-vs-Cyl (*p* = 0.262) in (**c**).

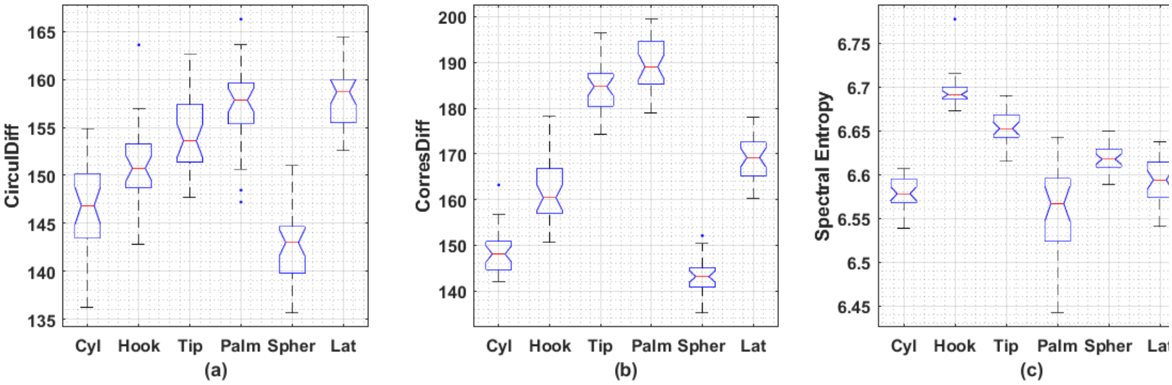

Figure S7. Comparisons of male one, channel two. Cases failed to pass Wilcoxon rank sum tests (2/45, confidence level is set to be 0.05): Palm-vs-Lat (p = 0.2838) in (**a**); Cyl-vs-Palm (p = 0.2643) in (**b**). Cases failed to pass Kruskal-Wallis test with Bonferroni's correction (17/45, confidence level is set to be 0.01): Spher-vs-Cyl (p = 1.000), Cyl-vs-Hook (p = 0.288), Hook-vs-Tip (p = 0.306), Tip-vs-Palm (p = 1.000), Tip-vs-Lat (p = 0.296), Palm-vs-Lat (p = 1.000) in (a); Spher-vs-Cyl (p = 1.000), Cyl-vs-Hook (p = 0.062), Hook-vs-Lat (p = 1.000), Lat-vs-Tip (p = 0.017), Tip-vs-Palm (p = 1.000) in (b); Palm-vs-Cyl (p = 1.000), Palm-vs-Lat (p = 1.000), Cyl-vs-Lat (p = 1.000), Lat-vs-Spher (p = 0.273), Spher-vs-Tip (p = 0.087), Tip-vs-Hook (p = 0.329) in (**c**).

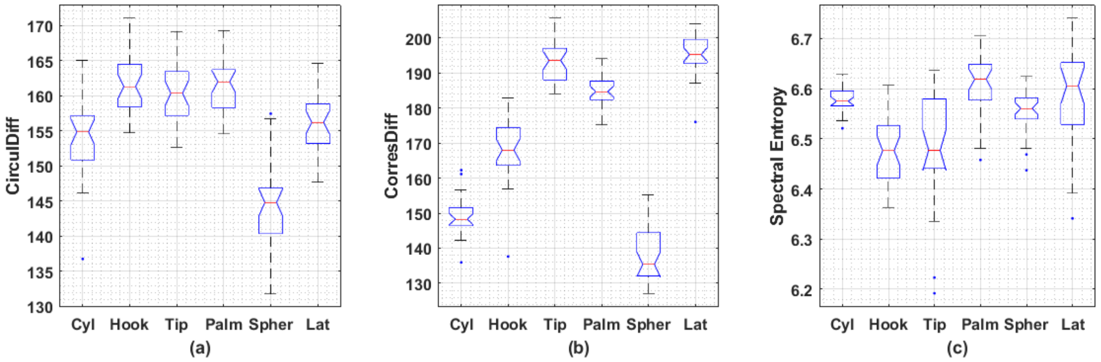

Figure S8. Comparisons of male two, channel one. Cases failed to pass Wilcoxon rank sum tests (8/45, confidence level is set to be 0.05): Cyl-vs-Lat (p = 0.0993), Hook-vs-Tip (p = 0.3329), Hook-vs-Palm (p = 0.9705), Tip-vs-Palm (p = 0.4553) in (**a**); Tip-vs-Lat (p = 0.0724) in (**b**), Cyl-vs-Lat (p = 0.1715), Hook-vs-Tip (p = 0.5997), Palm-vs-Lat (p = 0.5895) in (**c**). Cases failed to pass Kruskal-Wallis test with Bonferroni's correction (23/45, confidence level is set to be 0.01): Spher-vs-Cyl (p = 0.030), Cyl-vs-Lat (p = 1.000), Lat-vs-Tip (p = 0.113), Lat-vs-Palm (p = 0.011), Tip-vs-Palm (p = 1.000), Tip-vs-Hook (p = 1.000), Palm-vs-Hook (p = 1.000) in (a); Spher-vs-Cyl (p = 1.000), Cyl-vs-Hook (p = 0.275), Hook-vs-Palm (p = 0.120), Palm-vs-Tip (p = 0.210), Palm-vs-Lat (p = 0.024), Tip-vs-Lat (p = 1.000) in (b); Hook-vs-Tip (p = 1.000), Hook-vs-Spher (p = 0.039), Tip-vs-Spher (p = 0.786), Tip-vs-Cyl (p = 0.012), Spher-vs-Cyl (p = 1.000), Spher-vs-Lat (p = 0.438), Spher-vs-Palm (p = 0.012), Cyl-vs-Lat (p = 1.000), Cyl-vs-Palm (p = 0.781), Lat-vs-Palm (p = 1.000) in (**c**).

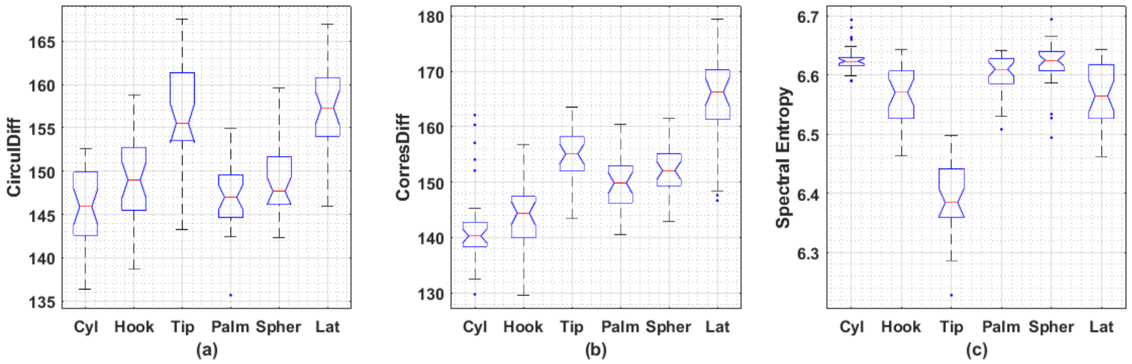

Figure S9. Comparisons of male two, channel two. Cases failed to pass Wilcoxon rank sum tests (8/45, confidence level is set to be 0.05): Cyl-vs-Palm (p = 0.3953), Hook-vs-Palm (p = 0.0933), Hook-vs-Spher (p = 0.6520), Tip-vs-Lat (p = 0.6843), Palm-vs-Spher (p = 0.1669) in (**a**); Cyl-vs-Hook (p = 0.0724) in (**b**), Cyl-vs-Spher (p = 0.9000), Hook-vs-Lat (p = 0.9941) in (**c**). Cases failed to pass Kruskal-Wallis test with Bonferroni's correction (20/45, confidence level is set to be 0.01): Cyl-vs-Palm (p = 1.000), Cyl-vs-Spher (p = 1.000), Cyl-vs-Hook (p = 0.548), Palm-vs-Spher (p = 1.000), Palm-vs-Hook (p = 1.000), Spher-vs-Hook (p = 1.000), Tip-vs-Lat (p = 1.000) in (a); Cyl-vs-Hook (p = 1.000), Cyl-vs-Palm (p = 0.016), Hook-vs-Palm (p = 0.113), Palm-vs-Spher (p = 1.000), Palm-vs-Tip (p = 0.165), Spher-vs-Tip (p = 1.000), Tip-vs-Lat (p = 0.160) in (b); Hook-vs-Lat (p = 1.000), Hook-vs-Palm (p = 0.618), Lat-vs-Palm (p = 1.000), Palm-vs-Spher (p = 1.000), Palm-vs-Cyl (p = 0.968), Spher-vs-Cyl (p = 1.000) in (**c**).
